# Poster Session II - A283 IMPROVEMENT OF BOWEL URGENCY WITH ADVANCED INFLAMMATORY BOWEL DISEASE THERAPIES: A SYSTEMATIC REVIEW AND META-ANALYSIS.

**DOI:** 10.1093/jcag/gwaf042.283

**Published:** 2026-02-13

**Authors:** N S Rai, P Patel, P Belesiotis, Y Yuan, N Narula, C Ma, M Cino, A N Sasson, V Jairath, P Tandon

**Affiliations:** McMaster University, Hamilton, ON, Canada; Nova Southeastern University, Fort Lauderdale, FL; University of Toronto, Toronto, ON, Canada; Western University, London, ON, Canada; McMaster University, Hamilton, ON, Canada; University of Calgary, Calgary, AB, Canada; University Health Network, Toronto, ON, Canada; University Health Network, Toronto, ON, Canada; Western University, London, ON, Canada; University Health Network, Toronto, ON, Canada

## Abstract

**Background:**

Inflammatory bowel diseases (IBD), including ulcerative colitis and Crohn’s disease, are chronic conditions that significantly affect quality of life. Bowel urgency is a particularly disruptive symptom of IBD, which is often underreported in clinical trials.

**Aims:**

This systematic review and meta-analysis aimed to examine the effects of IBD therapies on bowel urgency, focusing on the degree and durability of improvement.

**Methods:**

MEDLINE, Embase, and Cochrane databases were searched in December 2024 for studies that reported a bowel urgency outcome for IBD therapies. Only studies reporting absence of BU as a quantitative, binary outcome were included in the meta-analysis. A subgroup analysis was also performed by IBD subtype (Crohn’s disease vs ulcerative colitis). Risk ratios with 95% confidence intervals were reported.

**Results:**

Forty-four studies were included, including 29 randomized controlled trials and 15 post-hoc studies of RCTs representing a total of 8 therapeutic agents. There was significantly improved likelihood of BU remission across all induction (risk ratio 1.77, 95% confidence interval 1.51-2.08) and maintenance (risk ratio 2.40, 95% confidence interval 1.54-3.73) therapies, compared to placebo, with no major differences amongst the anti-interleukin-23 agents (Risankizumab, Mirikizumab and Guselkumab) and JAK-inhibitor therapy (Upadacitinib).

**Conclusions:**

The advanced IBD therapies studied produce rapid and sustained improvement in bowel urgency remission across mechanisms of action. The degree of bowel urgency improvement was similar between agents, potentially due to their action on similar inflammatory pathways. Areas for future research include investigation of bowel urgency outcomes with other IBD therapies, exploration of underlying mechanisms of action and greater standardization for measuring bowel urgency through validated scores.

**Funding Agencies:**

None

